# Hierarchically Structured Role-Playing Simulation as a Tool for Promoting Soft Skills in Veterinary Undergraduates

**DOI:** 10.3390/ani15111638

**Published:** 2025-06-03

**Authors:** Alejandro Perez-Ecija, Antonio Buzon-Cuevas, Adelaida De Las Heras, Francisco J. Mendoza

**Affiliations:** 1 Department of Animal Medicine and Surgery, Campus Rabanales, University of Cordoba, 14014 Cordoba, Spain; vetbuzon@gmail.com (A.B.-C.); fjmendoza@uco.es (F.J.M.); 2 Veterinary Teaching Hospital, Campus Rabanales, University of Cordoba, 14014 Cordoba, Spain

**Keywords:** leadership, role-playing, teamwork, veterinary education

## Abstract

Soft skills such as client-oriented communication, leadership, and teamwork skills are essential for veterinary clinicians. However, they are rarely addressed in the academic curriculum of most veterinary schools. Role-playing has been previously used to train and evaluate communication skills in veterinary students, but leadership and teamwork are difficult to implement in this methodology. In this study, we designed a novel role-playing activity where groups of undergraduate veterinary students faced simulated pre-planned clinical consultations portraying different roles in a hierarchically structured group (leader, two veterinarians, and one client). We tested the performance of the participants before and after receiving a focused lecture on soft skills. While the overall performance of the students in veterinary roles was significantly better after the lecture, the number of mistakes related to leadership did not significantly vary. Students rated the role of leader consistently lower than the rest. Participants considered that this novel design was useful to train and evaluate these competences. Hierarchically structured role-playing is a suitable tool to improve soft skills in veterinary students, allowing for self-learning and training in a controlled environment.

## Simple Summary

Veterinary clinicians are required to correctly communicate with pet owners, as well as lead and work in complex and interdisciplinary teams. These non-clinical competences are not the primary focus of veterinary education and are not easy to evaluate or train. In this study, a role-playing simulation where students portray different roles in a hierarchical structure was designed. We tested the performance of the students before and after receiving a lecture about these specific soft skills. Students performed significantly better after the lecture, although problems portraying the leader role remained. Students had an excellent reception of this activity and considered that it was highly useful.

## 1. Introduction

Soft skills are a series of non-technical professional competences covering areas such as social and communication competences, business management, entrepreneurship, leadership, and empathy [1]. Veterinary clinical consultation is a prime example of a setting where these skills are ubiquitously practiced and routinely challenged. Proficiency in these abilities is highly valued and can significantly improve the employability of new graduates [2,3]. Training in these competences has also been linked to higher job satisfaction and financial success in clinical veterinarians [4]. Among these soft skills, effective client-oriented communication, teamwork, and leadership skills are one of the most important for veterinary clinicians. All these competences are listed as “Day One Skills” by the European Association of Establishments for Veterinary Education (EAEVE), and regarded as fundamental for veterinarians [5]. Despite the overall importance of these soft skills, and prior to this simulation training event, undergraduate students in the Veterinary School of the University of Cordoba lacked any theoretical or practical training in them.

Effective communication during the veterinary consultation improves client satisfaction and adherence, contributes to better treatment compliance and outcomes, enhances professional well-being, and reduces the number of malpractice claims [6,7,8]. One of the most complex aspects of the veterinarian–client–patient interaction is the necessity of a shared decision-making process, able to harmonize the professional’s scientific knowledge and preferences with clients’ requirements and priorities [9]. Proper client-oriented communication is also critical during the delivery of bad news (death notifications, discussion of euthanasia, etc.), where clinicians should show empathy and compassion in a highly stressful situation [10].

Teamwork skills (effective professional communication, managing conflicts, etc.) are also fundamental for veterinary clinicians. Many veterinarians are employed in large facilities with a mix of colleagues with different knowledge, experience, and responsibilities, each one of them with sometimes divergent priorities, convictions, and goals [11]. Moreover, interactions with other disciplines (veterinary technicians/nurses, physical therapists, farriers, etc.) could further complicate the net of hierarchical relationships and communicational barriers [12]. Well-adjusted veterinary teams with a clear role understanding and open and healthy communication between their members are linked to higher job satisfaction, less propensity to burnout, and fewer medical errors [11,13,14].

Veterinarians are also commonly required to lead multidisciplinary teams. A competent veterinary leader should inspire and motivate the entire team, provide a shared vision and direction, be able to solve internal conflicts, distribute workload, and give valid feedback [15,16].

Role-playing (simulated veterinary consultations) has been previously used to teach communication skills to veterinary students [17,18,19]. With this methodology, different case scenarios can be created in order to portray future challenges and situations that will be routinary for the student. Since the simulations are performed in a safe and supportive environment, the level of insecurity and anxiety of the students is usually minimized, facilitating more effective learning without fear of making mistakes or mistreating any pet owner or animal. However, scarce reports of role-playing activities focused on teamwork or leadership skills can be found in the veterinary literature [20,21], and, to the authors’ knowledge, none of them were centered around a simulated veterinary consultation.

Therefore, the objectives of this study were as follows: (a) to create a novel veterinary consultation role-playing activity with a hierarchical structure, (b) to evaluate client-oriented communication, teamwork, and leadership skills in veterinary undergraduates using this new role-playing activity; (c) to study the variations in the students’ performance after receiving a short lecture/training in these soft skills; and (d) to determine the participants’ self-awareness about their proficiency in these soft skills.

## 2. Materials and Methods

### 2.1. Participants and Study Design

Undergraduate students from the third, fourth, and fifth year of the Veterinary School of the University of Cordoba participated in this study. The activity was voluntary and lacked any impact on the final qualifications of the participants. The authors explained the activity to the interested students, who were then randomly divided into groups of 4 students each. Dates for each phase of the activity were agreed with every group.

The activity consisted of three different phases:

- Phase 1: Initial role-playing. This simulation was performed in the facilities of the Veterinary Teaching Hospital of the University of Cordoba (Cordoba, Spain). Duration: 34–35 min. Dates for this phase were agreed with each group.
- Phase 2: Lecture about soft skills and their application to veterinary clinical settings. This part was carried out in a classroom with a maximum of 40 attendees (a total of 5 sessions were scheduled). Duration: 90 min. This phase was conducted once all of the participants had completed phase 1.
- Phase 3: Second role-playing after the lecture. This second simulation was performed in the same facilities as phase 1. Duration: 34–35 min. Dates for this phase were agreed upon with each group after every participant completed phase 2.

The total time of the activity (phases 1, 2, and 3) was 158–160 min, distributed on three different days.

### 2.2. Role-Playing Activities (Phases 1 and 3)

#### 2.2.1. Roles and General Activity Design

**Figure 1:**
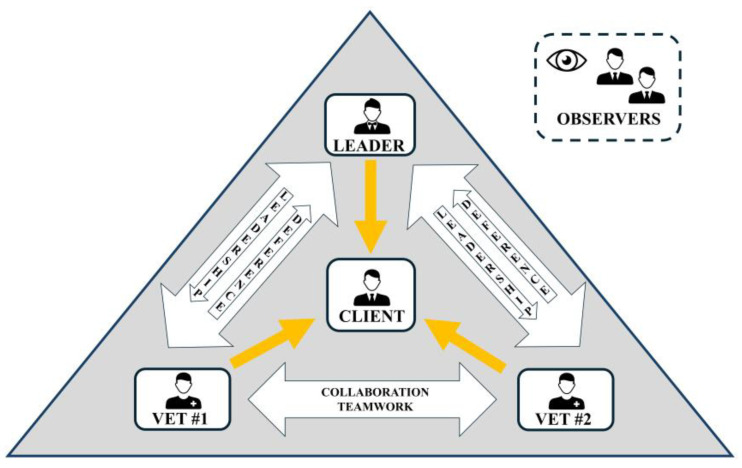
Schematic representation of the hierarchical structure of the role-play activity, displaying the relationships between different roles.

Participants within each group were randomly assigned a different role: client, leader, veterinary clinician 1 (vet #1), and clinician 2 (vet #2). Students kept the same role in the phases 1 and 3. Figure 1 was provided to the students prior to the activity in order to clarify the overall hierarchical relationship between roles, although no further explanations about the responsibilities or functions of each role were provided (looking for each group to be free to settle or explore them).

During the entire simulation, at least two authors were always present in the room, acting as observers. All of the observers were trained veterinary clinicians with decades of experience in the Veterinary Teaching Hospital of the University of Cordoba and private practices. All of the observers have implemented training sessions about soft skills, such as the one described below in Section 2.3.

Observers remained silent, taking notes, and evaluating the performance of the different clinical roles. No feedback was provided to the students about their performance during the entire experiment. The observers only intervened when each scenario was finished.

The activity consisted of a simulated veterinary clinical consultation (i.e., a dog with recurring vomits, a cat with ocular problems, etc.), which was subdivided into three scenarios. Brief intermissions (2 min) before scenario 1 and between scenarios were set up to allow the participants to rest and prepare their roles. Each scenario was limited to 10 min to promote time management.

In order to avoid repetitions, a total of 115 different cases were created with various species (cats, dogs, horses), clinical signs (provided to the client in scenario 1), final diagnosis (provided to veterinarians and leader in scenario 2) and treatment choices (provided to veterinarians and leader in scenario 2). Each group received different cases and cases were different between phase 1 and phase 3.

Before the simulation, the authors encouraged the participants to interact freely with other roles based on their understanding of each role’s responsibilities and functions. Clients were allowed to expand their given prompts (e.g., name or breed of the imaginary pet, monetary constraints, etc.), as long as the information in notes and prompts was maintained. During the simulation, a designated area (simulated back office) of the consulting room was established in case any participant wanted to communicate privately with other roles. In order to avoid distractions and bioethical considerations, no real animals were used in the simulations.

#### 2.2.2. Scenario and Prompt Description

Before every scenario, and depending on their role, each participant was given a series of prompts (Table 1).

The first scenario was focused on meeting a new client for the first time. For each simulated consultation, notes with information about the imaginary sick pet were provided to the student acting as a client, compiling basic information about the animal (species, age, sex, clinical signs, etc.).

The second scenario was designed to evaluate the shared decision making with the client, as well as the interactions between the leader and the veterinary team during a conflict. Prior to this scenario, specific notes were provided to vet #1 and vet #2 outlining the final diagnosis of the sick animal and their respective preferred choices for the treatment of this disease. In order to create a conflict, a blinded dichotomous decision was made, for example, vet #1 prefers a surgical approach versus vet #2 prefers medical treatment. Brief information about cost, efficiency, risks, and scientific back-up for each option was included in the notes. Once the veterinary team reached a consensus on how to discuss these options with the client, it was emphasized that they had to present them to the client.

The third scenario was focused on the ability of the veterinary members to communicate bad news (the death of the sick pet) to the client, deal with the client’s reactions, and work as a team during a stressful situation.

### 2.3. Lecture About Soft Skills (Phase 2)

The participants received a lecture (1.5 h) about soft skills based on available literature between phase 1 and phase 3 simulations. Briefly, the following topics were covered:

- Stages of the veterinary consultation as described by the Calgary–Cambridge observation guide (CCOG): preparation, initiation, gathering information, explanation/planning, and closure [22].
- Verbal and non-verbal communication in veterinary practice: communication strategies for veterinarians, categories of non-verbal behavior, how to shape spaces, how to address mixed messages, etc. [23].
- The six-step SPIKES protocol for delivering bad news in veterinary clinical settings [24].
- Shared decision making and effective client communication: the 4-E model for client communication [25].
- Teamwork-oriented skills in the veterinary setting: task management, workload distribution, dependability, and delegation [21].
- Leadership in veterinary clinics: creating a shared vision, consensus creation, and conflict management [11,16,26].

### 2.4. Evaluation of the Role-Playing Simulations and Project Suitability

The observers present in each group rated the performance of all the clinical roles (leader, vet #1, and vet #2) by counting the incidents (problems related to veterinary soft skills) in each simulation (both in phase 1 and phase 3). Observers should reach a consensus between them about the final number and type of incidents for each group.

Incidents were classified into three categories: client-oriented communication errors, leadership mistakes, and teamwork failure (Table 2). Client-oriented communication incidents were based on simplified versions of the observable behaviors from the CCOG, the 4-E model for client communication, and the SPIKES methodology, in each of the scenarios respectively [22,24,25]. Incidents related to teamwork and leadership skills were based on simplified versions of the characteristics of a successful veterinary team and the expected skills of an ideal veterinary leader [21,26]. Results were anonymous (no track was maintained about the student committing each incident).

The performance of each clinical role was also evaluated by the students themselves. Once phase 1 was completed, every participant was asked to rate (0—bad- to 10—perfect-) the performance of each other role during the simulation (including him/herself, except for the client). This rating was asked again once phase 3 was finished. This evaluation was performed with an anonymized online questionnaire specific to each role using a Moodle platform based on the internal servers of the University of Cordoba. Prior to answering the questionnaires, written consent was obtained from all the participants. Data were processed in line with EU regulations (Regulation (EU) 2018/1725 of the European Parliament and of the Council of 23 October 2018).

In addition, students were also asked to assess (0–10) the ability of the activity to promote each soft skill, and provide an overall rating of the project considering its objectives and design.

### 2.5. Statistical Analysis

Data from the evaluation (number of incidents per group and distribution per category) and questionnaires (phase 1 and phase 3 roles’ cross-evaluations and auto-evaluations, overall rating of the activity, and ability to promote each soft skill) were compiled and anonymously studied. Normality was assessed by a Kolmogorov–Smirnov test. Results are expressed as mean ± standard deviation (SD) and median. The Tukey’s Hinges test was used to calculate the median and percentiles.

Differences between Phase 1 (pre-lecture) and Phase 3 (post-lecture) incidents per group, as well as the number of incidents per category and group and the results of each role cross- and auto-evaluations, were compared using a paired t-test or a Wilcoxon test, depending on the normality.

Statistical analyses were performed using a statistical software (GraphPad Prism 9, San Diego, CA, USA), and values with *p* < 0.05 were considered significant.

## 3. Results

### 3.1. Participants and Simulations

A total of 52 groups initially performed the phase 1 simulations, although only 50 groups (*n* = 200 students) completed the entire activity. Two groups were unable to attend the lecture or perform the second simulation. Thirty-one participants were students from the third year of the course, with 113 and 56 being fourth- and fifth-year students, respectively. A total of 76% (152) were female and 24% (48) were male students.

### 3.2. Total Count of Incidents and Incidents per Soft Skill (Evaluation by the Authors)

**Figure 2:**
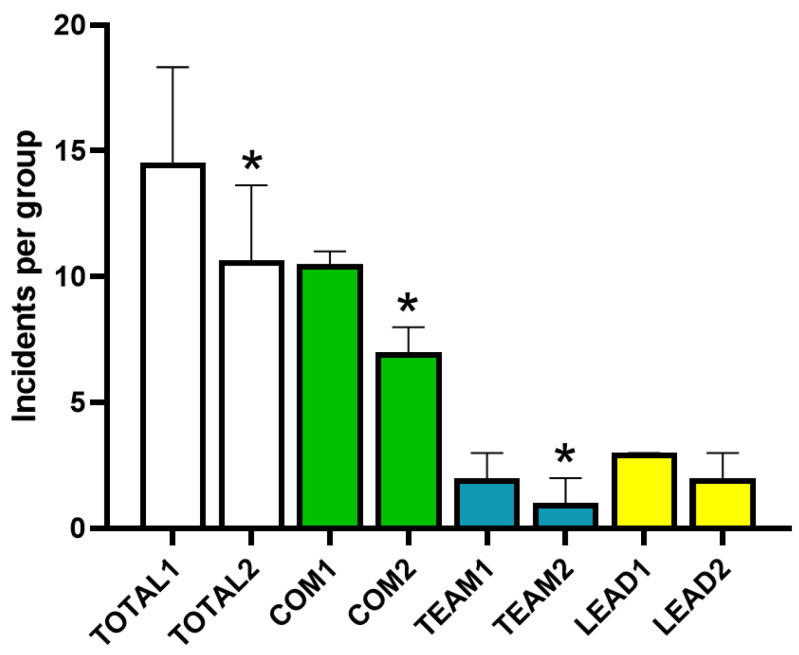
Average total incidents in phase 1 (pre-lecture) and phase 3 (post-lecture) per group of students and average number of incidents for each soft skill evaluated during the activity. Columns represent the mean and the bar is the standard deviation. TOTAL1, total number of incidents per group in pre-lecture simulations; TOTAL2, total number of incidents per group in post-lecture simulations; COM1, client-oriented communication mistakes in pre-lecture simulations; COM2, client-oriented communication mistakes in post-lecture simulations; TEAM1, teamwork mistakes in pre-lecture simulations; TEAM2, team-work mistakes in post-lecture simulations; LEAD1, leadership errors in pre-lecture simulations; LEAD2, leadership errors in post-lecture simulations. * *p* < 0.05 vs. pre-lecture.

Phase 3 showed a significantly (*p* < 0.0001) lower total count of incidents (mean 10.64, median 10) compared to phase 1 (14.54, median 14) (Figure 2). Client-oriented communication (10.26 and 7.14 respectively, *p* < 0.0001) and teamwork (1.84 and 1.26, *p* = 0.001) incidents were significantly lower in phase 3 than in phase 1, whereas no differences (*p* = 0.19) were observed for incidents related to leadership between both phases (2.44 and 2.24, respectively) (Figure 2).

### 3.3. Role Evaluation (Evaluation by the Students)

All of the students (those playing as clients, leaders, and vets) rated veterinarians higher than the leader (Table 3). Leaders were indeed failed (evaluation <5) by veterinarians (both phases) and themselves (phase 1) (Table 3).

When the rating of each role was compared between phase 1 and phase 3, both veterinarians (*p* < 0.0001) and leaders (*p* = 0.01) showed improvement in the later simulation according to the clients. Leaders also perceived a significant improvement in the performance of veterinarians (*p* < 0.0001) and themselves (*p* < 0.0001); while students acting as veterinarians only perceived this improvement in leaders (*p* = 0.005), and not themselves (Table 3).

### 3.4. Overall Activity Evaluation by the Students

The activity and its capability to reach its objectives was rated with 8.39 ± 1.2 (median 8) points. No differences were found in this rate between roles.

According to the questionnaires, the experience was more able to promote client-oriented communication (8.80 ± 1.2, median 9) than teamwork (7.38 ± 1.7, median 7) or leadership skills (6.91 ± 2.0, median 7). No differences were found in these ratings between roles.

## 4. Discussion

The use of hierarchically structured role-playing consultation simulations can be considered an effective tool to evaluate and train multiple non-clinical soft skills in a safe, controlled, and judgment-free environment in veterinary undergraduates. A significant improvement in client-oriented communication and teamwork skills was noted in participants after receiving targeted instruction on best practices concerning these competences.

Compared to previously reported experiences [17,18,19,27], our design is innovative due to the creation of a hierarchical structure, where students portray different roles with dissimilar functions and responsibilities. This approach allowed us to expand the teaching and evaluation capabilities of role-playing to teamwork and leadership skills which have been historically difficult to implement [20,21].

One possible weakness of our design is the use of students as simulated clients. In the last years, the use of actors (professional or amateur) in this role has been reported in veterinary studies [10,17]. Although this option shows some clear benefits (actors can portray a wider range of emotions, are prepared to better resemble verbal and non-verbal archetypes, and can perform a standardized role), it is usually expensive, requiring higher organizational efforts and intensive schedule management [17,18]. We chose to use students as simulated clients in part because of these reasons, but also because this factor did not significantly alter the ability of role-playing to improve students’ performance in communication skills in a previous study [17].

Soft skills have been usually recognized as difficult to evaluate and train in a standardized setup [28]. Previous authors have reported the use of videotapes and an interaction analysis system in order to evaluate the performance of veterinary students in role-playing activities [8]. In our case, we could not use this methodology due to concerns with data protection. Thus, we relied on an anonymized evaluation of students’ performance by the authors (observers) using a list of incidents. Client-oriented communication incidents were based on the CCOG, the 4-E model for client communication, and the SPIKES methodology; all of them widely used consultation models and methodologies adapted for veterinary education [8,22,25,29]. Incidents related to teamwork and leadership skills were more difficult to define, since scarce literature is available in veterinary medicine. We based our selection on simplified versions of the characteristics of a successful veterinary team (excellent teamwork rubric) previously described by Hanley and colleagues [21], and the expected skills of an ideal veterinary leader [26]. While this list of incidents was aimed to standardize the procedure, some degree of subjectivity cannot be dismissed due to the human nature of the evaluators. We tried to reduce this factor by demanding at least two observers to be present in each simulation, who should reach a consensus about the final number and type of incidents.

The average number of incidents encountered per group in phase 1 was high (average 14.54; maximum possible 20). This finding was expected due to groups acting in this phase blindly, the novelty of the activity, and the lack of previous knowledge about these skills. The majority of these initial mistakes were related to communication (average 10.26; maximum possible 12), which was more probably explained by this category being the largest and also the activity being heavily oriented towards client interaction. Leadership incidents were also proportionally high (average 2.24; maximum 4). This could also be related to the blind nature of this first phase, where participants lacked any guide to properly develop their functions and responsibilities inside the hierarchical structure.

While role-playing activities are highly valuable to promote soft skills, their potential can be enhanced when they are combined with previous specific training. This has been previously proved in veterinary education in regard to client communication, teamwork, and leadership [2,10,21,30]. Moreover, students who have previously received any type of formation in these competences are usually more aware of their relevance in the profession and tend to be more objective during self-assessment [2]. Our results agree with these previous reports, since a significant improvement in the performance of the participants was observed after a short focused lecture was provided. This effect was mostly noted in client-oriented communication skills, which could be explained by students being aware of the proper structure of the veterinary consultation and being able to easily solve some of the incidents (i.e., failure to introduce themselves, non-verbal language-related incidents, etc.). However, no significant differences were found in leadership incidents, which continued to be proportionally elevated. This could be related to these skills being inherently difficult to portray by the students and/or the lecture being too short to properly clarify the intrinsic singularities of this role. On the other hand, this could also be related to our checklist of leadership-related incidents not being sufficiently detailed or comprehensive. From the authors’ point of view, the lack of a validated checklist to measure and assess teamwork and especially leadership skills signifies the need for further research on this topic, maybe in collaboration with organizational psychology experts.

Self-assessment was used in this experience in order to evaluate the students’ perception of their own (and their peers) performance. Although this tool is not objective, it is widely applied in these types of studies in veterinary medicine [14,21]. During phase 1, the role of leader was consistently rated as the worst performing among the clinical ones (even by students acting as leaders themselves). This result could signify a misunderstanding of the responsibilities and duties of this role and/or an inability to properly enact them during this first phase of the activity. Negative attitudes towards leading figures cannot be discarded. Nonetheless, an overall improvement in the performance of the leader was noted by all of the students after the second simulation, which demonstrates that the lecture was beneficial for these participants.

Based on the results from the questionnaires provided to the participants, the activity was rated as excellent and regarded as a highly valuable and useful tool to promote communication, teamwork, and leadership skills.

The present study has some limitations. First, since students performed the simulation twice, previous exposure to the activity and gained experience could have had some influence on the improvement in skills during phase 3, independently of the effects of the training session. Interestingly, this confounding factor could be discarded when considering leadership skills, where no significant difference was found between simulations. Future studies using control groups and different variations in the schedule of phases (e.g., groups repeating the experience without any training or groups performing their first simulation after the training) should be necessary in order to confirm our findings. Second, groups were randomly created, mixing third-, fourth-, and fifth-year students, who are likely to have different experiences and exposure to consultations. Although none of them had any specific training in soft skills during their curriculum, a more homogenous grouping could have been interesting to avoid this effect and determine differences between years. Third, although every group had different simulated cases, we cannot be completely sure that students performed in a completely blind way (we did not control interactions between students of different groups outside of the experiment). This could have been possible with a lower number of participants and limiting the duration of each phase, but we prioritized a large number of students participating. Finally, the lack of incidents based on CCOG, the 4-E, and SPIKES methods, and teamwork and leadership ideal characteristics could not perfectly reflect a good veterinary clinical performance. Nonetheless, it is worth noting that clients consistently rated both veterinarians and leaders higher in phase 3 (where fewer incidents were present).

While the overall reception of this project was excellent, and students acknowledged its utility in promoting soft skills, its ability to encourage leadership skills was deemed as suboptimal. Thus, this type of experience could be implemented as a valid practical evaluation mechanism to accredit the acquisition of soft skills in undergraduates after receiving a proper theoretical formation. Future modifications and revisions of this design should be investigated in order to enhance its impact on leadership skills.

## 5. Conclusions

Hierarchically structured role-playing simulations are a useful tool to train and evaluate communication, teamwork, and leadership skills in veterinary undergraduates. The performance of students in these simulations was significantly improved after receiving theoretical training focused on these competences. Leadership skills appear to be inherently difficult to perform for veterinary students and a longer (a more focused) training course could be necessary for them to understand the responsibilities and nuances of this role. Participants rated this new methodology as highly valuable and acknowledged its utility in promoting soft skills in veterinary students.

## Figures and Tables

**Table 1:** Compilation of prompts given to each role during the activity.

**Scenario 1. The Beginning of the Consultation**	
**Role**	**Prompts**
*Client*	Check notes about your imaginary pet. You can make up any information about you or your pet not specifically included in them. This is your first time in this Hospital.
*Vets*	Receive this new client, establish initial rapport, and identify the reasons for the consultation. Consider collecting all of the client’s concerns about the pet.
*Leader*	Prepare your team to receive this new client. Distribute the workload between the team members according to your own criteria.
**Scenario 2. Proposing and Considering Two Conflicting Options**	
**Role**	**Prompts**
*Client*	Several options are going to be discussed for your imaginary pet. You can make up any consideration (economical, ethical, previous experience, knowledge, etc.) about them.
*Vets*	In face of the diagnosis (check notes), there are 2 different options for this case. Based on your background, experience, and knowledge, you have a preferred one (check notes). Consult with your colleagues about the better option. Once you have reached a consensus, this should be discussed with the client.
*Leader*	In face of the diagnosis (check notes), there are 2 different options for this case. Try to collect the preferences of each of your team members and reach a consensus prior to talking to the client. Remember that you are the leader of this team.
**Scenario 3. Delivering Bad News**	
**Role**	**Prompts**
*Client*	The veterinary team is going to present you with some news about your pet.
*Vets*	The pet has died unexpectedly during a routine process. This must be reported to the client.
*Leader*	The pet has died unexpectedly during a routine process. This must be reported to the client. Remember that you are the leader of this team.

**Table 2:** List of incidents evaluated during the activity.

**Client-Oriented Communication Errors**
Fail to welcome/greet the client or attend to his/her comfort
Fail to introduce him/herself or any member of the team
Fail to ease the situation and establish a relaxed environment (lack of previous chit-chat)
Inadequate verbal expressions or inadequate non-verbal clues (i.e., lack of eye contact, inadequate posture and position or facial expression, incorrect use of tone, awkward spacing, etc.)
Fail to identify the primary reason for the consultation
Lack of open questions (avoiding the client to further express concerns and opinions)
Neglect of secondary/additional concerns and perspectives by the client
Lack of active listening skills (interrupting the client, directing their responses, etc.)
Fail to provide a structured planning during the consultation (steps, times, etc.)
Inappropiate shared decision making with the client (fail to consider the client’s perspective and preferences, use of bias, directing the decision, etc.)
Inadequate delivery of bad news (lack of warning shots, excessive jargon, etc.)
Lack of empathy
**Teamwork Mistakes**
Inappropiate teamwork (overshadowing other vet roles, absenteeism)
Contradicting other veterinary roles in front of the client
Lack of coordination with the rest of the team
Inadequate teamwork during the conflict (fail to listen actively, fail to give and receive feedback to/from other members of the team)
**Leadership Failures**
Inadequate distribution of workload by the team leader (or carelessness)
Fail to motivate the members of the team (support, empathy, validation)
Inadequate leadership during decision making (fail to promote a consensus, authoritarianism, avoiding responsibilities, inappropiate conflict solving, etc.)
Fail to assume responsibilities by the leader during the delivery of bad news (absenteeism during this step or excessive delegation)

Each incident was counted with 1 point.

**Table 3:** Rating of each role by participants.

	Phase 1			Phase 3		
Client	Vet (both)		Leader	Vet (both)		Leader
	6.85 ± 2.2 (7)		6.64 ± 1.8 (7)	8.04 ± 1.3 (8) ^1,2^		7.06 ± 1.7 (7) ^1^
Leader	Vet (both)		Self	Vet (both)		Self
	6.06 ± 1.5 (6) ^2^		4.62 ± 1.4 (5)	6.54 ± 1.1 (7) ^1,2^		5.42 ± 0.8 (5) ^1^
Vet	Self	Cross	Leader	Self	Cross	Leader
	6.47 ± 1.2 (7) ^2^	5.99 ± 1.6 (6) ^2^	4.41 ± 1.9 (4)	6.71 ± 0.8 (7) ^2^	6.24 ± 1.3 (6) ^2^	4.97 ± 1.7 (5) ^1^

Data are expressed as mean ± standard deviation (SD) and median. Cross, evaluation of the other student acting as vet; Self, self-evaluation of the student in this role; Vet, veterinarian. ^1^ *p* < 0.05 vs. same role in phase 1. ^2^ *p* < 0.05 vs. leader in the same phase.

## Data Availability

The data presented in this study are available upon request to the corresponding author.

## Abbreviations

The following abbreviations are used in this manuscript:

CCOG	Calgary-Cambridge Observation -Guide
MDPI	Multidisciplinary Digital Publishing Institute
